# Investigating factors for using insurance apps by customers: a study of a developing country

**DOI:** 10.1007/s42786-022-00046-9

**Published:** 2022-11-09

**Authors:** Quoc Trung Pham, Huynh Ngoc Diem Nguyen, Sanjay Misra, Abhavya Misra

**Affiliations:** 1 Ho Chi Minh City University of Technology (VNU-HCM), Ho Chi Minh City, Vietnam; 2 Ostfold University College, Halden, Norway; 3 Guild Insurance Group, Brandon, Canada

**Keywords:** Insurtech adoption, Insurance application, Technology acceptance, Risk, Trust, Vietnam

## Abstract

With the fast development of insurtech, many researchers are doing research in understanding customer behavior regarding the adoption of insurance applications. However, there is a lack of research on the subject in developing countries such as Vietnam. The primary purpose of this article is to develop a predictive model for the intention to use the insurance application of individual customers in Vietnam. In this research, the quantitative research method is conducted based on survey data collected in Ho Chi Minh City. Data are tested using various methods, such as Cronbach’s Alpha, EFA, Pearson correlation test, and multiple linear regression. From the analysis results, some factors positively influencing the intention to use insurance applications are identified, including Trust, Ease of Use, and Usefulness. However, the impact of Risk on Usefulness is not confirmed based on the sample data. From the analysis results, some managerial implications are made to improve the intention to use insurance applications of customers in Vietnam.

## Introduction

After 20 years of economic development, insurance market in Vietnam is developing quickly. Till the end of 2020, there are 10 million people who bought life insurance (about 10% of the whole population). Currently, there are 70 enterprises working in the insurance industry, including life insurance, non-life insurance, health insurance, and other mediating forms of insurance service. Since 2020, during the Covid-19 period, the estimated scale of the world insurance market is 6.1 trillion USD (decreased 2.8% in comparison with 2019). However, in Vietnam, the insurance market still increases to nearly 185 thousand billion dongs (an increased 15% in comparison with 2019). In, the revenue of non-life insurance is about 57.1 thousand billion dongs (increased 8% in comparison with 2019), of life insurance, is about 127.6 thousand billion dongs (increased 19.6% in comparison with 2019) [3].

With the fast development of IT and the Internet, insurance companies are racing for digital transformation and applying more insurtechs to their products and services. In 2018, the total insurance fee through insurtechs was about 187 billion USD, approximately 4% of the total fee of world insurance. The predicting number will be more than 400 billion USD in 2023 (about 7% of the total fee of world insurance) [7].

In Vietnam, the insurance market is in the same trend. Some big insurance companies, such as: Bao Viet life insurance, Prudential Vietnam, Dai-Ichi Life, are implementing various insurance applications. These applications are not only playing the role of a digital assistant to consult their customers but are also being used for optimizing the service processes and increasing customers’ experiences.

Besides, according to the Vietnam Social Insurance report [19], there are about 16.17 million Vietnamese purchased social insurance, of which 15 million people are compulsory. Regarding health insurance, there are 87.77 million people participated. At the same time, Vietnam Insurance Company encouraged the usage of VssID, an integrated insurance application, for connecting with users in social and health insurance, and making it convenient for them in using the service. However, although there are more than 3.7 million downloads, the real number of patients who used the VssID application is still low. According to Hanoi Medical University [22], the reason could be in the habit of Vietnamese users, especially the old and the poor people, who are not familiar with mobile applications and still use paper-based insurance cards.

Another survey by Vietnam Report [24], showed that there are some difficulties for an insurance company in applying mobile applications and in the digital transforming process, such as lack of infrastructure, lack of expertise, low application quality, the concern of security problems, lack of training… Although the insurtech brings a lot of benefits for employees, agents, and customers, the acceptance of Vietnamese users of the insurance application is limited. Moreover, the number of empirical studies on the adoption of insurtech in Vietnam is still low. Therefore, research to assess the influence of factors on the intention to use the insurance app of individual customers in Vietnam is needed.

In summary, the key objectives of this paper are (1) to explore the key factors influencing the users’ adoption of insurance applications in Vietnam; and (2) to suggest management implications for increasing the users’ acceptance of insurance applications. The structure of this research is organized as follows: Sect. 2 summarizes the main concepts and literature review; Sect. 3 proposes the research model; Sect. 4 presents the key research results, and Sect. 5 describes the discussion and main conclusions.

## Background and related work

### Insurtech in Vietnam

Insurtech is a mix between “Insurance” and “Technology”. Insurtech refers to the application of technology innovations to optimize operations and enhance the efficiency of the insurance business model. Some examples of Insurtech include mobile applications, wearable devices, compensation tools, online insurance agreement processing, customers’ data collection and analysis [2].

Currently, there are 5 groups of Insurtech related to traditional insurance services, including (1) Big data, AI, and analytics; (2) Digital based insurance applications (web, mobile, social media); (3) Internet of things; (4) Cyber insurance; (5) Health and medical insurance [12].

In Vietnam, more and more insurance companies are utilizing websites or mobile applications in increasing customers’ service and differentiating their products. For example, FWD [9], applied a full online payment service, where customers can sign the insurance agreement completely on their tablet or smartphone. Another example is PTI [20], which collaborated with INSO Vietnam, to develop an INSO application, which allows customers to buy insurance packages and request compensation without using any paper-based form. Bao Viet [1], company integrated a solutions management information system on the mobile platform with the name Baoviet Direct, which allows customers to easily purchase, monitor, manage their rights, and request for a compensation.

In 2020, the Vietnamese government encouraged the application of ICT in the insurance industry. Vietnam Insurance Company (a state-own enterprise) implemented 19 online public services at levels 3 and 4. All of these online services will reach a level at the end of 2021. Moreover, a mobile application of health and social insurance (VssID) is going to deploy during 2021–2022, which brings many benefits to insurance service users and the whole society [21].

### Users’ acceptance: TAM and UTAUT

There are two popular theories in exploring the impact factors of users’ acceptance of new technology including (1) the Technology Acceptance Model (TAM) and (2) the Unified Theory of Acceptance and Use of Technology (UTAUT).

The TAM, suggested by Davis et al. [6], contains two important factors influencing the acceptance of an information system: the perception of usefulness and ease of use. Where the perceived usefulness is affected by the perceived ease of use.

The UTAUT, suggested by Venkatesh et al. [25], contains certain determinants which include the expectation of performance, effort, social impact, and supporting conditions. This model also mentions the impact of demographic variables (age, sex, experience, and willingness) on the end-user intention and behavior.

### Relevant researches

Some relevant research on the adoption of fintech/ insurtech are presented in Table 1.

**Table 1:** Works on the adoption of fintech/insurtech

Authors	Focus	Main findings
Mai et al. [14]	Behaviors of purchasing life insurance Location: Vietnam	Impact factors: product accessibility, subjective norm, attitude, risk perception, finance knowledge, buying intention, and behavior. Results found that all hypotheses were supported. In which, finance knowledge has the strongest impact, and the subjective norm has the weakest impact
Meyliana et al. [18]	Adoption of fintech services Location: Indonesia	Impact factors: trust, perceived risk, usefulness, ease of use, attitude, and intention to use. Results found that trust and usefulness have a positive impact, but perceived risk and attitude don’t have a significant impact
Hu et al. [13]	Adoption intention of Fintech services for bank users Location: China	Impact factors: trust, user innovation, government support, brand image, perceived risk, attitude, ease of use, usefulness, and intention to use. Results found that trust has a strong impact on the attitude toward fintech, but perceived risk and ease of use have no significant impact
Chong et al. [4]	Adoption of Fintech service Location: Malaysia	Impact factors: perceived ease of use, perceived usefulness, social influence, personal innovativeness, security concern, perceived enjoyment, and intention to adopt. Results found that all hypotheses were supported. In which, enjoyment has the strongest impact and social influence has the weakest impact
Chuang et al. [5]	Adoption of Fintech service Location: Taiwan	Impact factors: brand and service trust, perceived usefulness, perceived ease of use, attitude, and intention to use. Analysis results supported all hypotheses, in which perceived usefulness has the strongest impact

From the above literature review, TAM or UTAUT is used mostly as a base for exploring the adoption of the fintech/ insurtech. In this research, the TAM model is utilized as the foundation of the research model because it is used mostly and covered the significant factors impacting on the intention to use new technology like insurtech, including ease of use, usefulness, attitude, and the intent to use. Besides, in the insurance industry, 2 independent factors “trust” and “perceived risk”, which are confirmed to have strong impacts on the perceived usefulness of users toward insurtech, will be added to the proposed research model.

## Development of model and hypothesis statements

### Research framework

From the literature review, the TAM model is used as a base of the research. Besides, “trust” and “perceived risk” will also be added to the research model as mentioned in previous ones [13, 18].

**Fig. 1:**
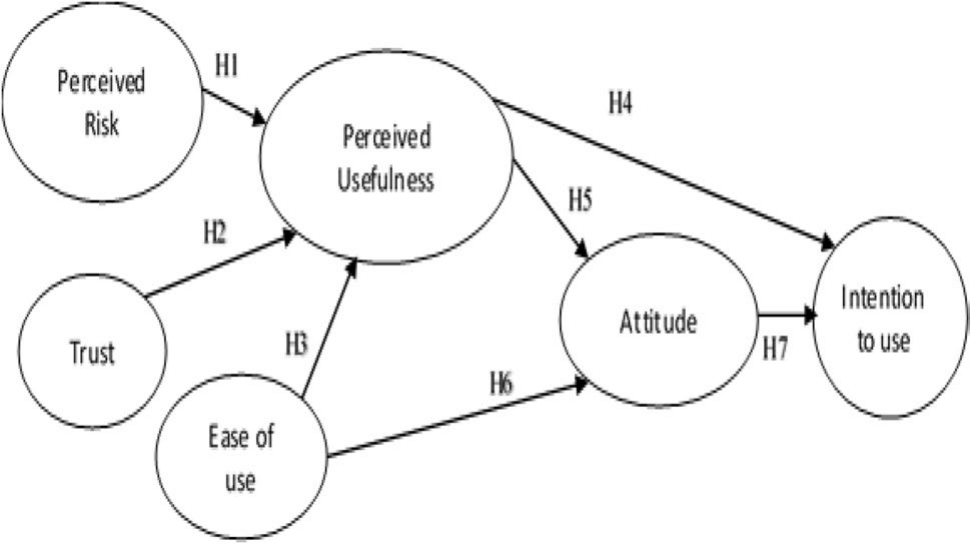
Research model

Our proposed research model is illustrated in the Fig. 1.

### Hypothesis statements

*Perceived risk* represents consumer uncertainty regarding loss or gains in a given transaction [17]. In this research, the perceived risk is the perception of customers about their loss or negative consequences of using insurtech. According to Zhang and Yu [26], risk perception also refers to risk perception in particular applications. Previous research [18] showed that perceived risk has an impact on perceived usefulness. So, the H1 hypothesis could be stated as follows:

#### H1

 Perceived risk has a positive impact on the perceived usefulness of insurance application users.

*Trust* is the willingness to respond to a partner with whom they have confidence in each other [15]. According to Morgan and Hunt [16], trust is at the heart of all interpersonal interactions. The trust exists only when one side has confidence in the trustworthiness and integrity of a trading partner. In this research, trust is an important factor influencing users of insurance apps. Based on Hu et al. [13], trust is found to be the most important factor impacting the perceived usefulness of users. So, the H2 hypothesis could be formulated as follows:

#### H2

 Trust has a positive impact on the perceived usefulness of insurance application users.

*Perceived ease of use* is the degree, to which a person believes that the use of a given system would be effortless [6]. The TAM model has shown that perceived ease of use has an impact on the perceived usefulness and attitude of users toward the new technology. In previous research [11, 13], there is a significant correlation between ease of use, usefulness, and attitude of users in the adoption of new technology. So, the H3 and H4 hypotheses could be summarized as follows:

#### H3

 Perceive ease of use has a positive impact on perceived usefulness of insurance application users.

#### H4

 Perceive ease of use has a positive impact on the attitude of insurance application users.

*Perceive usefulness* is the degree to which a person believes that using a particular system would improve their job performance [6]. According to Hansen [11], perceived usefulness has a significant positive effect on the attitude and behavioral intention of users. These linkages are also supported by other studies [5, 13]. So, the H5 and H6 hypotheses could be described as follows:

#### H5

 Perceive usefulness has a positive impact on the attitude of insurance application users.

#### H6

Perceive usefulness has a positive impact on the intention to use insurance application users.

*Intention to use* is defined as the degree of an individual’s willingness to use new technology [5]. Based on TAM, the attitude of users towards a new technology will determine their intention to use the technology. Previous research [13] has also confirmed such a relationship. So, the H7 hypothesis could be stated as follows:

#### H7

 Attitude has a positive impact on the intention to use insurance application users.

### Research process

The research is carried out as per following:

- Step 1: conduct a literature review, propose the research model, and develop the draft measurement scales.
- Step 2: the draft questionnaire is used to interview 10 users of the insurance application and 5 developers/ managers. The primary objective is to verify the clarity and correct errors on the provisional scales. As a result, the final questionnaire is created and used for the next step.
- Step 3: a survey will be conducted to collect data from a variety of insurance companies in Vietnam. The sample should include ≥ 200 individual customers. This information will then be used to verify reliability, consistency, and hypothesis. Some statistical tools to test the model include Cronbach alpha, EFA, Pearson correlation, and Multiple-regression analysis.
- Step 4: post-result interviews with various stakeholders will be made to discuss the results, and to give recommendations for increasing the intention to use the insurance application in Vietnam.

### The original measurement scales

All measurement scales are developed based on 5 levels of Likert scales. The original scales of perceived ease of use (PE), perceived usefulness (PU), attitude (ATT), and intention to use (INT) are from Chuang et al. [5]; perceived risk (PR) and trust (TR) are from Hansen et al. [11]. After primary qualitative research step, there are some small revisions of the original scales (e.g., correcting spelling mistakes, rephrasing, removing some duplications, adding the insurance context). The final questionnaire could be seen in the appendix.

## Analysis results

### Descriptive statistics

Collection of data using the convenience sampling approach. The final questionnaires were scattered using various forms, such as Google Docs, E-mail, Insurance related fan-page, and paper copies to respondents who purchased an insurance package in Vietnam. There were 209 questionnaires received. In which 166 samples are valid after the cleaning phase. The sample descriptive statistics by demographics is summarized in the following Table 2.

**Table 2:** Sample statistics by demographics

Category	Values	Frequency	Percentage
Gender	Male	90	54.2
	Female	76	45.8
Age	< 20 years old	3	1.8
	20–29 years old	80	48.2
	30–39 years old	61	36.7
	≥ 40 years old	22	13.3
Job	Students	16	10
	Public sector manager/employee	39	23
	House-worker	5	3
	Private sector manager/employee	99	60
	Financial consulting	6	4
	Freelancer	1	1
Income per month	< 10 million VND	26	15.7
	10–20 million VND	109	65.7
	> 20 million VND	31	18.6

The sample descriptive statistics by the key factors of the research model is summarized in Table 3.

**Table 3:** Descriptive statistics by key factors

Main factors	N	Min	Max	Avg	Stdev
Perceived ease of use (PEU)	166	1.00	5.00	4.26	0.82
Perceived usefulness (PU)	166	1.00	5.00	4.15	0.91
Trust (TR)	166	1.00	5.00	3.97	0.86
Perceived risk (PR)	166	1.00	5.00	3.25	1.20
Attitude (ATT)	166	1.00	5.00	4.20	0.79
Intention to use (INT)	166	1.00	5.00	4.17	0.80

### Cronbach’s alpha analysis

Cronbach’s alpha analysis is used to test the reliability of the scales. According to Hair et al. [10], the Cronbach alpha coefficient and the item-total correlation should be ≥ 0.6, and ≥ 0.3 respectively. Table 4 shows that the Cronbach alpha coefficients of all scales are greater than 0.6. Therefore, all scales can be used for the EFA analysis.

**Table 4:** Cronbach’s alpha results

Factors	Alpha	Item-total correlation	#Item removed
Perceived ease of use (PEU)	0.880	0.489–0.767	0/6
Perceived usefulness (PU)	0.889	0.507–0.806	0/8
Trust (TR)	0.856	0.627–0.821	0/3
Perceived risk (PR)	0.915	0.748–0.882	0/3
Attitude (ATT)	0.818	0.545–0.708	0/4
Intention to use (INT)	0.923	0.805–0.857	0/4

### Exploratory factor analysis (EFA)

According to EFA results, if the KMO coefficient = 0.851 (> 0.5) then EFA can be used. Table 5 presents that there were 6 factors extracted, and the extraction variance was 64.53%. Once 4 variables are removed (low loading factor coefficients or loaded in several factors), there were 24 remaining variables grouped into 6 factors. The result had total extraction variance = 67.77% (> 50%), KMO = 0.896 (> 0.5), Bartlett test was significant (Sig. < 0.05). So, it could be used for the next step. The EFA results could be found in Table 5.

**Table 5:** The exploratory factor analysis result (*PAF* promax rotation)

	1	2	3	4	5	6
INT1	0.899					
INT2	0.883					
INT3	0.866					
INT4	0.691					
ATT2		0.691				
ATT4		0.624				
ATT1		0.605				
PU2			0.796			
PU8			0.782			
PU3			0.769			
PU7			0.725			
PU6			0.717			
PU5			0.683			
PU4			0.647			
PEU3				0.841		
PEU4				0.803		
PEU5				0.739		
PEU6				0.725		
PEU2				0.650		
PR2					0.965	
PR1					0.920	
PR3					0.788	
TR2						0.888
TR3						0.798

### Pearson correlation

Pearson analysis is conducted for assessing the correlation between independent variables and dependent variables. The analysis results could be summarized as follows:

- Perceived usefulness has a significant correlation (sig. < 0.05) with trust, and perceived ease of use. However, the correlation with risk is insignificant (sig. = 0.871 > 0.05), so the impact of risk on perceived usefulness should be re-evaluated in the regression model.
- Attitude has a significant correlation (sig. < 0.05) with the perceived usefulness and perceived ease of use.
- Intention to use has a significant correlation (sig. < 0.05) with attitude and perceived usefulness.

### Multiple regression analysis

#### Model 1: risk, trust, perceived ease of use → perceived usefulness

They were running regression analysis for testing the hypothesis about the impact of independent variables (PR, TR, PEU) on the dependent variable (PU). Adjusted R square is 0.343 showed that independent variables generated 34.3% of the variances of the dependent variable. According to ANOVA table, significant value of F-test is 0.00 < 0.05. Therefore, the regression model is suitable. Table 6 summarizes the regression analysis for model 1.

**Table 6:** The regression analysis for model 1 (PEU, PR, TR → PU)

Model		Unstandardized coeff.		Standardized coeff.	t	Sig	Collinearity statistics	
		B	Std. error	Beta			Tolerance	VIF
1	Constant	1.302	0.336		3.873	0.000		
	PEU	0.452	0.081	0.420	5.576	0.000	0.702	1.425
	PR	0.043	0.040	0.068	1.051	0.295	0.957	1.044
	TR	0.202	0.060	0.258	3.361	0.001	0.677	1.477

Dependent variable: PU

According to the above table, H1 (PR → PU) is rejected because sig. = 0.295 (> 0.05). H2 (TR → PU) and H3 (PEU → PU) are confirmed because their sig. values < 0.05. The rejection of H1 could be explained by the maturity of technologies for insurance app helps to reduce the uncertainty of customer in doing a transaction, and lower the impact of perceived risk on the usefulness. Besides, insurance customers focused more on the risk of insurance products than on the risk of insuretech.

#### Model 2: perceived ease of use, perceived usefulness → attitude

Adjusted R square is 0.352 showed that independent variables generated 35.2% of the variances (PEU, PU) of the dependent variable (ATT). According to ANOVA table, significant value of F-test is 0.00 < 0.05. Therefore, the regression model can be considered suitable. Table 7 summarizes the regression analysis for model 2.

**Table 7:** The regression analysis for model 2 (PEU, PU → ATT)

Model		Unstandardized coeff.		Standardized coeff.	t	Sig	Collinearity statistics	
		B	Std. error	Beta			Tolerance	VIF
1	(Constant)	1.469	0.293		5.013	0.000		
	PEU	0.309	0.076	0.309	4.093	0.000	0.691	1.447
	PU	0.345	0.070	0.371	4.921	0.000	0.691	1.447

^a^Dependent variable: ATT

According to the above table, H5 (PU → ATT) and H6 (PEU → ATT) are confirmed because their sig. values < 0.05.

#### *Model 3: perceived usefulness, attitude* → *intention to use*

Adjusted R square is 0.626 showed that independent variables generated 62.6% of the variances (PU, ATT) of the dependent variable (INT). According to ANOVA table, significant value of F-test is 0.00 < 0.05. Therefore, the regression model is suitable. Table 8 summarizes the regression analysis for model 3.

**Table 8:** The regression analysis for model 3 (PU, ATT → INT)

Model		Unstandardized coeff.		Standardized coeff.	t	Sig	Collinearity statistics	
		B	Std. error	Beta			Tolerance	VIF
1	Constant	0.146	0.249		0.584	0.560		
	PU	0.197	0.059	0.188	3.323	0.001	0.706	1.417
	ATT	0.759	0.064	0.676	11.943	0.000	0.706	1.417

^a^Dependent variable: INT

According to the above table, H4 (PU → INT) and H7 (ATT → INT) are confirmed because their sig. values < 0.05. In which, attitude has the strongest impact on the intention (beta = 0.676). Therefore, using consultants to persuade customers and change their attitude is very important for increasing the intention to use insuretech.

Moreover, the regression results of the three models showed that there was no collinearity because the VIF coefficients were low (< 2). Besides, these models are suitable with other criteria of the multiple regression model (standardized residuals follow the normal distribution). So, these results are applicable.

## Discussion, implications, and conclusions

### Results discussion

The analysis result presented that customers' intention to use insurance applications in Vietnam is affected by their attitude (0.6767) and perceived usefulness (0.188). At the same time, the perceived usefulness is affected by the ease of use (0.420) and trust (0.258). Besides, the attitude was affected by the perceived service (0.371), and the ease of use (0.309). These results are partially similar to previous studies by Meyliana et al. [18] and Hu et al. [13]. But the weight of impact is somehow different. The impact of trust and perceived usefulness is confirmed in all results, but the effect of perceived risk is not confirmed either. The reason could be the insurance customers didn’t care much about the risk of using insuretech because they focused more on insurance products. However, in Meyliana et al. [18], the attitude has no significant impact, but, in this research, the attitude has the strongest impact on the intent to use insurance applications. This difference could be explained by the cultural aspect of Vietnamese customers, who have more emotional behaviors. In Hu et al. [13], ease of use has no significant impact, but, in this research, ease of use positively impacts the attitude toward insurance applications. In Vietnam, the use of insure app is still at the early phase, so, the ease-of-use is very important in changing customers’ attitude and behavior.

From these results, perceived ease of use and trust are 2 independent variables that can be considered for improving the attitude and intention to use insurance applications of customers. Therefore, insurance companies should pay attention to designing their friendly and easy applications for learning and using. Besides, increasing the reliability and secure policy will also help improve the intention to use.

### Managerial implications

From these results, several implications for managers to increase the user attitude and to improve the intention to use insurance applications of individual customers could be suggested as follows:

- Trust is an important factor impacting perceived usefulness, influencing users’ attitudes and intention to use insurance applications. Therefore, increasing customers’ trust is a possible solution. If customers believe that their personal information is secured, they will use the insurance application more. The insurance company should have a public data collection and protection policy (e.g. GDPR of European countries). It will help increase the reliability of the application and improve the intention to use it for customers.
- Perceived usefulness is considered one of the most important mediating factors impacting the attitude and intention to use insurance applications. The main features of insurance applications must be included to increase the perceived usefulness, such as information searching, process reduction, and time-saving… Therefore, insurance companies should pay attention to developing more innovative applications, more convenient utilities, and integrated functions to improve their applications' usefulness.
- Ease of use is also important for increasing users’ attitudes toward insurance applications. Therefore, the insurance company should make it easy for their customer by simplifying processes, providing user manuals and training clips… Online course and chat bot could also help to improve the ease of use and to encourage self-study process of customers. The easier the application is designed, the more positive the customers have toward the application.
- Besides, insurance companies need to improve their insurance apps to be more safe, operationally efficient, and competitive. They should train their agents and customers and create effective communication between insurers and customers. These solutions may help generate a positive attitude of customers toward insurance products and insuretechs, which will increase the intention to use these apps.

### Conclusions

Generally, based on the TAM model and previous research, this research proposed a model for assessing the intent to use insurance applications of individual clients in Vietnam. Some main factors were examined, including trust, perceived risk, perceived usefulness, perceived ease of use, attitude, and the intent to use.

Based on 166 valid samples collected from insurance customers in HCMC (Vietnam), the research hypotheses were tested. The analysis results found that (1) trust and perceived ease of use have positive impacts on perceived usefulness; (2) perceived ease of use and perceived usefulness have direct impacts on attitude toward insurance application; and (3) perceived usefulness and attitude have positive impacts on the intention to use insurance application. Only one rejected hypothesis of the result is the impact of perceived risk on perceived usefulness.

Given this outcome, the managers of insurance companies should have certain policies to enhance the customers’ trust, perceived usefulness, and ease of use of their insurance applications. It will help to improve customers’ attitude and finally increase their intent to use the insurtech.

However, there are some limitations of this paper, including (1) The limited sample size, (2) The lack of evaluation the impact of some other factors, such as: social influence, financial knowledge, etc. Therefore, some directions for future researches could be as follows: (1) Expand the size and reach of data samples; and (2) Evaluate the impact of some new factors on the intention to use the insurtech.
